# Supplementary material for: Validation of a novel handheld lactate analyzer reveals concentration-dependent bias compared with a laboratory reference device
Source: Biol Open. 2026 Apr 22;15(4):bio062543. doi: 10.1242/bio.062543 (PMC13148469; doi:10.1242/bio.062543)
Supplement: Supplementary information [file biolopen-15-062543-s1.pdf]

Table S1. 95% CIs of Bland–Altman analysis.

Available for download at
https://journals.biologists.com/bio/article-lookup/doi/10.1242/bio.062543#supplementary-data

Dataset 1. Collected paired samples.

Available for download at
https://journals.biologists.com/bio/article-lookup/doi/10.1242/bio.062543#supplementary-data

Biology Open • Supplementary information
